# Novel Strategy to Prevent Cancer Metastasis with Metastasis-Regulating miRNAs Found in Extracellular Vesicles Secreted from Stemness-High Cells

**DOI:** 10.3390/cancers17172734

**Published:** 2025-08-22

**Authors:** Mikako Saito, Hideaki Matsuoka

**Affiliations:** 1 Department of Biotechnology and Life Science, Tokyo University of Agriculture and Technology, Tokyo 184-8588, Japan; 2 Bioresource Laboratories, Tokyo University of Agriculture and Technology, Tokyo 184-8588, Japan; mhide@cc.tuat.ac.jp

**Keywords:** stemness-high cells, Nanog, iPS cells, extracellular vesicles, metastasis

## Abstract

Preventing metastasis and recurrence after cancer treatment remains a challenge. Extracellular vesicles (EVs) have long garnered attention as tools for vaccination. To develop high-performance vaccines, there has been an ongoing search for high-performance miRNAs and high-performance EVs as resources. In recent years, stemness-high cells have been reported to represent valuable resources for EVs, offering a level of performance not previously achieved. A differential miRNA analysis was performed between metastasis-suppressive EVs and metastasis-promoting EVs to predict miRNAs specific to metastasis regulation. These newly identified miRNAs were expected to show good performance in metastasis suppression. It is noticeable that these miRNAs seem to be categorized differently from the cancer-associated miRNAs that have been extensively studied to date. In order to further develop vaccine therapy, it is widely recognized that continuing to explore methods for further enhancing the metastasis-suppressive potential of EVs and mRNAs is a fundamental and urgent task. Significant progress has been made through the discovery of stemness-high cells as new EV resources and the results of miRNA exploration focused on metastasis regulation. This review aims to address current challenges by presenting relevant, up-to-date information and providing insights that may lead to new discoveries.

## Simple Summary

Extracellular vesicles (EVs) secreted by cancer cells were previously believed to contribute to metastasis promotion. However, recent studies have shown that EVs secreted by stemness-high cells, such as stem cells and stemness-intensified cancer cells, may exert significant suppressive effects on cancer metastasis. These suppressive effects are speculated to be mediated by immune cells, and therefore, elucidating the functional roles of various contents of EVs in regulating immune cells is recognized as an urgent challenge. A current key aim is to identify high-performance miRNAs from EVs and clarify their functional roles and target genes. This review aims to contribute to achieving this objective by providing up-to-date, relevant information.

## 1. Introduction

Cancer immunotherapy research using extracellular vesicles (EVs) is currently undergoing rapid development. EVs include exosomes (30–150 nm), microvesicles (150–1000 nm), and apoptotic bodies (1–5 μm). Many landmark reports have been published regarding exosomes, but in recent years, there have been a number of reports suggesting that EVs more widely, not limited to exosomes, represent an extremely promising resource for use in developing substances for cancer therapy [1,2,3,4,5]. In addition, although limited, vaccine therapy using EVs is also being carried out [6]. The main reason why EVs are attracting attention from the perspective of cancer treatment is that EVs are expected to offer a trump card for use against the problems of metastasis and recurrence caused by small numbers of residual cancer cells, which are difficult to solve using standard treatments such as surgery, anticancer drugs, and radiotherapy. In other words, EVs can be used as vaccines and delivered systemically in a cell-free manner and may be effective in attacking minute amounts of cancer cells that are difficult to locate.

However, EVs contain a complex and diverse group of molecules, and it is extremely difficult to select only those components that are effective in suppressing metastasis. In addition, the mechanism behind metastasis suppression is not the same for each molecule, and it is difficult to establish optimal conditions for the simultaneous application of these molecules. Even if an optimal set of molecules can be prepared, it is also important to improve the technologies used to deliver them stably to the relevant sites in the body [7,8].

Many recent reviews have concluded that the properties and roles of individual components in EVs need to be studied in more detail. For the development of vaccine therapy, it is widely recognized that continuing to explore methods of further enhancing the metastasis-suppressive potential of EVs and mRNAs is a fundamental and urgent task. Recently, it has been shown that EVs obtained from stemness-high cells may exert significant effects in metastasis suppression [3,9,10]. This strongly suggests that EVs from stemness-high cells could provide novel resources that offer better performance than any previously used. 

**Figure 1:**
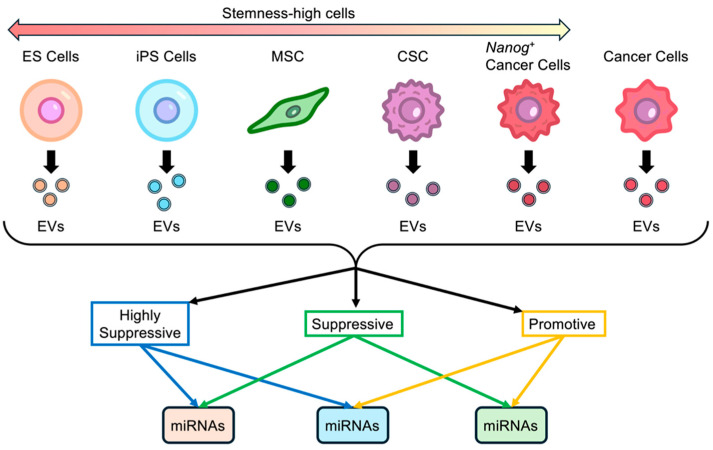
Stemness-high cells are EV resources with significant metastasis suppression effects. ES cells, embryonic stem cells; iPS cells, induced pluripotent cells; MS cells, mesenchymal stem cells; CS cells, cancer stem cells; *Nanog*^+^ cancer cells, *Nanog* overexpressing cancer cells.

There are no quantitative indicators for stemness, and therefore, there is no fixed definition of stemness-high cells. The term is used by the authors for convenience in contrast with cells that have differentiated into specific functions (Figure 1). Embryonic stem (ES) cells, which are undifferentiated cells, can be considered the most stemness-high cells. This is followed by induced pluripotent stem (iPS) cells, mesenchymal stem (MS) cells, and others. Cancer cells may be considered cells that have differentiated into specific functional cells. Cancer cells obtained by introducing a stemness-intensifying factor such as *Nanog* are considered to have more stemness-high than the cancer cells of the original wild cell line. Additionally, cancer stem (CS) cells are considered to have more stemness-high than cancer cells [11,12,13]. 

Differential miRNA analysis between metastasis-suppressive EVs and metastasis-promoting EVs may predict miRNAs specific to metastasis regulation. This analytical method is not new but is especially effective in identifying metastasis-regulating miRNAs that are differently categorized from the cancer-associated miRNAs that have been extensively studied to date.

The effects of EVs secreted by stemness-high cells on cancer metastasis, whether they are promotive or suppressive, might not necessarily be the same as was previously understood. These EVs may play more multifaceted roles than anticipated. There is a growing hope that we will be able to discover novel functional molecules such as miRNAs and their target genes that can be used to develop cancer metastasis therapies.

## 2. Activation of Immune Cells with EVs Derived from Highly Metastatic Cancer Cells

EVs secreted from cancer cells play a dual role in vivo. One role is the promotion of growth in cancer cells. EVs secreted by cancer cells are taken up by tissue cells, thereby introducing cancer cell information into those tissue cells. This creates a metastasis-inducing niche, an environment that promotes the engraftment and proliferation of cancer cells in the vicinity [14,15].

The other role is the inhibition of the growth of cancer cells. When EVs secreted by cancer cells are taken up by dendritic cells, the dendritic cells are activated to secrete EVs that can activate T cells and macrophages to transform them into anticancer cells. Elements of the acquired immunity system, such as T cells, selectively exhibit anticancer properties against the original cancer cells secreting the EVs that initially activated the dendritic cells. There is a memory effect, meaning that the anticancer activity is retained for a certain period of time [16,17]. On the other hand, macrophages of the innate immune system have also been reported to exhibit this memory effect [18,19,20]. Although nonspecific, this suggests the possibility that responses to what is recognized as foreign to the organism may be sustained and remembered for a certain period of time. 

Cancer cells encountered by immune cells during the evolution of the disease are those that have survived conventional treatment and are therefore likely to be highly malignant. In order for immune cells to exhibit sufficient anticancer activity against such cells, it is necessary to activate them using EVs derived from malignant cancer cells. Experimentally, metastatic potency represents a practical indicator of the malignancy of cancer cells. 

## 3. Is Achieving Zero Risk of Cancer Metastasis Possible? 

Multiple genes have been proposed as stemness-intensifying factors. Among them, the homeodomain transcription factor *Nanog* is the key factor for maintaining self-renewal and pluripotency characteristics. In humans, the Nanog protein, coded by the Nanog1 gene, consists of 305 amino acids and contains a conserved homeodomain that binds to DNA. There are ten pseudogenes (*NanogP2*-*P11*), with *NanogP8* being highly expressed in cancer cells [21]. In mice, however, no pseudogene has been reported. Nanog proteins are central regulators controlling both embryonic stem cells and cancer stem cells and are considered a marker of poor prognosis in many types of cancer.

Therefore, it was predicted that the overexpression of *Nanog* in cancer cells would increase the metastatic potential of the cancer and lead to the progression of malignancy. To demonstrate this effect of *Nanog*, mouse melanoma was selected as the test material, because there are several cell lines of the same lineage with different intensities of metastatic potential (B16F0, B16F10, B16BL6, etc.). These cell lines are considered to be useful as controls for the quantitative evaluation of changes in the intensity of metastatic potential. Another reason why mouse melanoma was selected was that it forms solid black metastatic colonies, which are relatively easy to analyze (in terms of number and volume) via microscopic measurements. As expected, the *Nanog*-overexpressing strain showed higher metastatic potential than the wild-type strain [22].

However, when the properties of EVs from the *Nanog*-overexpressing and wild-type strains were examined, unexpected results were obtained. The initial expectation was that EVs from any cell would be metastasis-promoting, with the stronger metastasis-promoting EVs being from *Nanog*-overexpressing cells (*Nanog*^+^F10-EVs). In fact, however, EVs from a wild strain F10 (F10-EVs) were metastasis-promoting, while the *Nanog*^+^F10-EVs were, contrary to expectations, metastasis-suppressive [9]. Colon cancer showed similar results. *Nanog*-overexpressing colon 26 (*Nanog*^+^colon26) had a higher metastatic potential than wild-type colon 26, while the EVs obtained from *Nanog*^+^colon26-EVs showed metastasis suppression [23]. 

**Figure 2:**
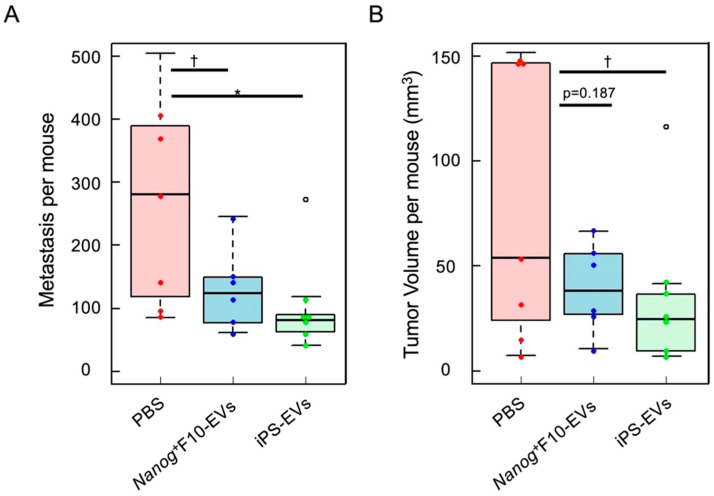
Metastasis-suppressive effects of *Nanog*^+^F10-EVs or iPS-EVs in mice [3]. Total number (**A**) and volume (**B**) of colonies in the liver and lungs. PBS n = 7, *Nanog*^+^F10-EVs n = 6, and iPS-EVs n = 7. (°) outlier, * *p* < 0.05, † *p* < 0.1.

It was considered that the metastasis-suppressive effect of EVs might be closely related to the fact that the cells that secreted EVs were stemness-high cells. Therefore, EVs secreted from cells with higher stemness may exhibit a more intense suppression effect than *Nanog*^+^F10-EVs. However, when embryonic stem cells and iPS cells were focused on, and EVs derived from mouse iPS cells (iPS-EVs) were applied to metastasis experiments using mouse melanoma (*Nanog*^+^F10), they showed even stronger metastasis suppression than *Nanog*^+^F10-EVs (Figure 2).

It was a mystery why iPS-EVs showed a suppressive effect on melanoma metastasis when there was no information about melanoma in the iPS-EVs. It was speculated that *Nanog*^+^F10-EVs and iPS-EVs exerted their suppressive effects through different molecular mechanisms. Therefore, it was expected that a combination of the different types of EVs may show additive effects and reduce metastasis to almost zero.

## 4. Candidates for Metastasis-Regulating miRNAs and Their Target Genes

miRNA sequencing analysis was performed on five types of EVs (*Nanog*^+^F10-EVs [S], F10-EVs [P], *Nanog*^+^colon26-EVs [S], colon26-EVs [P], and iPS-EVs [S]) ([S], metastasis-suppressive; [P], metastasis-promotive). miRNAs that changed significantly were determined using the differential analysis of [S]/[P] and are shown in Figure 2. Since iPS-EVs showed higher metastasis suppression than *Nanog*^+^F10-EVs[S], iPS-EVs[S]/*Nanog*^+^F10-EVs[S] differential analysis was performed. Based on the results, the miRNAs with the top 4–6 highest fold changes were predicted. After predicting the target genes of each miRNA using TargetScanMouse 8.0 at CWCS ≤ −0.4, the strength of functional association was analyzed using STRING for the predicted gene clusters, and the top genes with the strongest functional association were analyzed using Cytoscan/cytoHubba. The predicted top genes are candidates for hub genes, which represent the core of metastasis suppression signaling pathways.

STRING gene function information is compiled based on publicly available information on a gene. It provides a relatively comprehensive description of the functions of cells and tissues. Since particular emphasis was placed on information related to the immune system, keywords related to immune function were established, and we ranked the function-related intensities based on these keywords. Consequently, candidate metastasis-regulating miRNAs and target hub genes were predicted (Table 1).

## 5. Metastasis-Regulating miRNAs Are Different from Cancer-Associated miRNAs 

Cancer-associated miRNAs have been extensively studied [24]. In this review, the applications of miRNA-based diagnostic and therapeutic methods in cancer medicine are listed in a seven-page table citing 73 references. In total, 177 types of miRNA were identified in 17 types of cancer. In the cited literature, breast cancer and lung cancer were the most common, with 14 cases each. 

In the case of breast cancer-associated miRNAs [25], for example, five miRNAs are identified as “oncogenic miRNAs” that are involved in tumorigenesis, metastasis, and drug resistance, and four types of miRNAs are identified as “tumor-suppressive miRNAs” that are involved in the suppression of tumor-related properties (Table 2).

Of the miRNAs in Table 1, however, only miR-19a-3p and miR-19b-3p were mentioned in the review [24], and no type of miRNA was common between those in Table 1 and Table 2, suggesting that there were very few previous studies concerning the miRNAs in Table 1. To further confirm the lack of studies on the miRNAs in Table 1, relevant studies in the literature were searched for through PubMed-NCBI (https://pmc.ncbi.nlm.nih.gov/). A search for “miR-21&cancer”, for instance, selected 43,979 articles. The average number of articles selected for five oncogenic miRNAs and four tumor-suppressing miRNAs in Table 2 was 22,709 and 20,390, respectively. In contrast, the average number of selected articles for five up-miRNAs and seven down-miRNAs in Table 1 was only 1434 and 1224, respectively. 

This indicates that metastasis-regulating miRNAs seem to form a small group, different from the large group of cancer-associated miRNAs. Furthermore, miRNAs assigned as metastasis-regulating miRNAs have only recently begun to attract interest as a research topic.

## 6. Recent Work on miRNAs Associated with Stemness-High Cells

Recent work on miRNAs’ roles in ES, iPS, and MS cells (including some miRNAs in EVs) is summarized in Table 3. An emphasis is placed on whether the miRNAs were tumor-promotive or tumor-suppressive and whether they were able to serve as indicators of the properties of tumor cells or immune cells.

### 6.1. ES Cells

Human embryonic stem (hES) cells are pluripotent cell lines established from internal cell masses harvested from human blastocysts. To investigate ES cell-specific miRNAs, miRNAs expressed in ES cells and embryonic cancer cells (EC cells) were comparatively analyzed. Of the 36 miRNAs identified via cDNA cloning, 8 were expressed in both ES and EC cells, while 7 were expressed only in ES cells [26]. The temporal changes in the expression of miRNAs during early differentiation from ES cells were investigated. The results showed that they could be classified into five clusters. Once a cluster with a high correlation with safety (free from cancer risk) was determined, the miRNAs expressed in that cluster would represent the next safety marker [27]. However, there have been no reports involving safety markers since then.

### 6.2. iPS Cells

For practical use in tissue regeneration, iPS cells are easier to use than ES cells. In such cases, the quality of the cells, including safety, is important. miRNAs are candidates for such indicators; one is miR-27b, which is highly conserved in vertebrates and involved in the regulation of various target genes in signaling pathways [49]. miR-27b also plays a role in the suppression of embryonic development in human iPS cells by acting antagonistically to the BMP signaling pathway [30]. Therefore, miR-27b must be downregulated to promote embryogenesis. Before differentiation, the maintenance of pluripotency is essential, but the selectivity and efficiency are important when differentiating in a specific direction. miRNAs involved in these cases are described in [31,33] and [32,34,35,37], respectively.

If EVs are to be used for practical purposes, then their mass preparation is required. An index consisting of ten miRNAs has been proposed for such a purpose [38].

A striking suggestion is that cancer cells could be rendered stemness-high via a novel method using miRNAs [36]. Four types of miRNAs were introduced into colon cancer cells, and ES cell-like cells were obtained, expressing undifferentiation marker genes of *Nanog*, *Oct3/4*, *SOX2*, and *Klf4*, as well as tumor-associated antigen 1–60. Previously, ES cell-like cells could also be produced via induction with four transcription factors (*Oct3/4*, *Sox2*, *c-Myc*, and *Klf4*) according to the conventional remodeling method. However, the expression of the oncogene c-*Myc* remained at a high level, which was a serious problem. The novel miRNA method solved this problem.

### 6.3. MS Cells

Mesenchymal stem cells (MS cells) can also be differentiated into various cell types. miRNAs are involved in promotion, regulation, and suppression in differentiation processes.

A suppressive role has been observed in osteogenesis. When MS cells were co-cultured with human periodontal ligament cells (HPL cells) for periodontal tissue regeneration therapy, bone formation was suppressed under the influence of fluid secretions from HPL cells. miR-299-5p was involved in this suppressive effect [39]. When MS cells were co-cultured with human gingival fibroblasts (HGFs), bone formation was also suppressed under the influence of humoral secretions from HGFs. In this case, miR-101-3p was involved in the suppressive effect [40].

miR-503 promoted vascular smooth muscle cell (VSMC) differentiation by inhibiting SMAD7 via the TGF-β pathway from upstream. In addition, miR-143 and miR-145 were upregulated by activated TGF-β1. On the other hand, miR-222-5p conversely repressed VSMC differentiation by downregulating ROCK2 and α-SMA [41]. This is an example of a situation in which promotion and suppression occur simultaneously. Numerous similar cases have been observed.

miR-200b-3p promoted the differentiation of MS cells into insulin-producing cells through interaction with the zinc finger E-box binding homeobox 2 (ZEB2) 3’ untranslated region (UTR) [42]. miR-145 promoted the differentiation of bone marrow MS cells into smooth muscle cells [43]. Osteogenic differentiation was promoted by miR-29b via the PTEN/AKT/β-catenin signaling pathway [44] and also regulated by miR-125b by targeting Cdfβ [45]. miR-34a activated alveolar differentiation by inhibiting TGF-βsignaling and activating the BPM pathway [46].

The idea of applying miRNAs as indicators of cell quality is exemplified by iPS cells. However, a similar method was applied to MS cells to determine the differentiation potential in a specific direction using the expression status of multiple miRNAs [47]. The ability of MS cells to differentiate into neurons was investigated to explore potential therapeutic tools for neurological diseases. After the induction of differentiation and conversion, miRNA expression was analyzed over time and classified into five clusters based on expression trajectories. It was found that each cluster contained unique miRNAs [48].

## 7. miRNAs in Innate Immune Cells

In future, when facing attacks from invading cancer cells in the absence of cancer cell information, it is considered that the main players will be those of the innate immune system, that is, macrophages and natural killer (NK) cells, and the involvement of dendritic cells (DCs) must also be considered. Therefore, we have summarized examples of the roles of miRNAs acting within macrophages, NK cells, and dendritic cells or from outside of each cell.

### 7.1. miRNAs Ccontributing to Macrophage Activation

There are many reviews on the involvement of miRNAs in macrophage function. With respect to data supporting the interaction between miRNAs and metabolism in macrophage inflammatory responses, the focus has been on key miRNAs and miRNA families, and furthermore, the impact of these networks on human disease has been discussed and summarized [50]. Another review comprehensively describes the molecular mechanisms of miRNAs in macrophage polarization, to analyze the potential value of regulatory pathways in the context of tumor and inflammation intervention therapies [51]. Some commentaries focus on specific diseases. For example, in asthma, there is an imbalance between classically activated macrophages (M1 cells) and selectively activated macrophages (M2 cells), with the latter predominating. Therefore, data on miRNAs known to correlate with specific human macrophage phenotypes and polarization, as well as their association with adult asthma, have been compiled [52]. Macrophages also play an important role in the progression of acute lung injury (ALI)/acute respiratory distress syndrome (ARDS) and exhibit different polar morphologies depending on the stage of disease progression. Therefore, an overview of miRNA expression in ALI/ARDS was presented, and the mechanisms and pathways by which miRNAs respond to macrophage polarization, inflammation, and apoptosis were introduced [53].

On the other hand, there is an interesting commentary on the homeostasis of the M1/M2 state of macrophages [54], which will be discussed below in detail. The polarization of macrophage M1/M2, which detects bacterial and viral motifs (pathogen-associated molecular patterns, PAMPs) in bacteria- and virus-infected tissues, increases tissue inflammation through cytokine production and kills bacteria and viruses. This inflammation-induced activation is referred to as the classically activated state or M1-like macrophages. In addition to PAMPs, the detection of inflammatory cytokines such as interferon gamma (INFγ) also results in M1-like macrophages. In contrast, the detection of proinflammatory cytokines such as interleukin 4 (IL-4) and IL-13 leads to the suppression of the inflammatory response, removal of cellular debris, and reconstruction of the extracellular matrix. This state is referred to as an alternative activation state or M2-like macrophages. Furthermore, if the M1 state persists, which may result in abnormal inflammatory activity, a feedback inhibition loop is activated to maintain homeostasis. Similarly, in the case of the M2 state, a feedback inhibition loop is activated to prevent excessive activity and maintain homeostasis. The function of macrophages is not simply polarized based on whether they are in the M1 or M2 state; rather, they have a fine-tuning function to maintain homeostasis in both states. miRNAs are mainly responsible for this fine-tuning function (Table 4).

The action of miRNAs is a challenging issue from a therapeutic point of view, because the target cell types differ in terms of their metastasis-inhibitory effects. miR-146 is an example of complicated regulation. miR-146 is a novel miRNA that is known to suppress the metastasis of cancer cells. The activation of the Jak-Stat signaling pathway (mainly Stat3) in cancer cells increases cancer cell survival and proliferation. In contrast, suppressor of cytokine signaling 1 (SOCS1) suppresses the Jak-Stat signaling pathway, so the activation of SOCS1 in cancer cells suppresses cancer cell growth. Since miR-146 can suppress SOCS1, miR-146 can potentially promote cancer cell growth. This process is complicated: when miR-146 is upregulated in cancer cells, SOCS1 (Down)/Jak-Stat (Up)/cancer cell proliferation (Up) is observed, which is counterproductive for metastasis suppression. In macrophages, however, SOCS1 suppresses the Cytokine/Toll-like receptor (TLR) signaling pathway. As a result, macrophage survival and proliferation are suppressed. Thus, increased expression of miR-146 in macrophages has a favorable effect on metastasis suppression: SOCS1 (Down)/cytokine, TLR (Up)/macrophage proliferation (Up). Therefore, when applying miR-146 in vivo, it must be delivered selectively to macrophages only. The high quality of miRNAs means that even if there are multiple target genes, none of them are guaranteed to have a negative effect on metastasis inhibition.

### 7.2. miRNAs Contributing to NK Cell Activation

In the human immune system, potent miRNAs and miRNA clusters have been discovered that play a pivotal role in the regulation of gene expression, termed immune miRNAs. Immune miRNAs exert a regulatory influence on both innate and acquired immune cells. They do not necessarily activate anti-tumor effects but may conversely blunt anti-tumor responses and create an environment inducive to tumor survival [55]. B7-H3 (a member of the B7 family of immune checkpoint molecules) is involved in T cell immune responses but has been newly shown to be involved in NK cell function, as well [56]. In mouse experiments, the inhibition of B7-H3 in ovarian cancer prolonged mouse survival. In vitro, miR-29c directly targeted and inhibited the expression of B7-H3, enhancing the anti-tumor effect of NK cells. However, in vivo, miR-29c/B7-H3 suppression did not improve the anti-tumor effect. There was a decrease in miR-29 family members (miR-29a, miR-29b, miR-29c) in deceased patients, which correlated inversely with B7-H3 expression in neuroblastoma patients. miR-29 family members promoted NK cell infiltration and activation and induced tumor cell apoptosis [57].

Natural Killer Group 2 Member D (NKG2D) is an important activation receptor for NK cells. UL16-binding protein 2 (ULBP2) is constantly expressed or elevated in cancer cells and serves as an important NKG2D ligand. The binding of ULBP2 and NKG2D initiates NK cell activation and the subsequent targeted elimination of cancer cells. Thus, enhanced expression of ULBP2 leads to a more efficient elimination of these cells by NK cells. The administration of resveratrol (RES) reduced intracellular miR-17-5p levels and increased ULBP2 expression, since ULBP2 was a target of miR-17-5p. As a result, the cytotoxic activity of NK cells was increased using RES [58].

The effects of miRNAs are not always as expected. In breast cancer (BC) tissue, miR-338-3p levels were significantly reduced. This trend was more pronounced in patients with advanced stages. To inhibit breast cancer cell survival, miR-338-3p was overexpressed. However, the axis of miR-338-3p (Up) in BC/ADAM17 secretion (Down)/granzyme B, CD16, and NKG2D (Down) inNK/NK cell function (Down)/BC survival (Up) ultimately resulted in an effect that increased BC cell survival [59].

An integrated miRNA and mRNA immune cell signature was constructed to predict survival in breast and ovarian cancer patients. miRNA expression-based immune cell signatures were constructed from the breast and ovarian cancer data sets of The Cancer Genome Atlas and combined with existing mRNA immune cell signatures. The constructed signatures enabled a more accurate depiction of prognostic and predictive immune cell subsets in both cancers [60].

### 7.3. miRNAs Involved in Dendritic Cell Function

Using miRNA array analysis, a representative miR-5119 was identified in breast cancer as a potential regulator of PD-L1 in dendritic cells (DCs). miR-5119 mimic modified DCs effectively restored the function of exhausted CD8+ T cells in vitro and in vivo and resulted in potent anti-tumor cellular immune responses, increased cytokine production, and decreased T cell apoptosis [61].

Cancer-associated fibroblasts (CAFs), a major component of the tumor microenvironment (TME), are known to modulate immune responses through the secretion of exosomes containing various growth hormones, miRNAs, and cytokines, but their effects on DCs were unknown. To confirm the effect of CAF-derived exosomes on DC maturation, DCs were cultured in the presence of a CAF-derived acclimation medium (CAFs-CM). The role of epigenetic regulators in maturation was assessed via the expression of miR-221, miR-222, miR-155, miR-142-3p, and miR-146a. Among them, an increased expression of miR-146a was observed, which was positively correlated with an increased expression of anti-inflammatory cytokines such as IL-6, IL-10, and TGF-β and a decreased expression of TNF-α (inflammation-inducing) [62].

DCs primed with exosomes rich in miR-155 inhibited tumor growth. Primed DCs significantly increased IL-12p70 and IFN-γ in serum and accelerated the differentiation, proliferation, and cytotoxic effects of helper T cells and cytotoxic T cells [63]. miR-155-overexpressing dendritic cells (DCs) were used to generate a DC vaccine. In mice transfected with this vaccine, an increase in effector T cells, the suppression of tumor growth, and a dramatic decrease in lung metastases were observed [64].

The miRNAs cited in this section are listed in Table 5.

## 8. Properties and Roles of Metastasis-Regulating miRNAs Demonstrated in Other Studies

According to a PubMed search, the number of papers dealing with metastasis-regulating miRNAs was extremely small. There was very little commonality between metastasis-regulating miRNAs (Table 1) and miRNAs of other categories (cancer-associated miRNAs and those cited in Section 6 and Section 7). Although there are few papers on these metastasis-regulating miRNAs, we have summarized their properties and functional roles as described in those papers (Table 6). In the case of research on cancer and disease, it seems that many miRNAs exacerbate the condition. In light of this situation, the remarkable effect of miRNAs in suppressing cancer metastasis seems to be an extremely rare phenomenon. This strongly suggests that the search for miRNAs contributing to the suppression of cancer metastasis is still insufficient. 

## 9. Conclusions

A systematic study focusing on metastasis-regulating miRNAs is important in facilitating the development of novel miRNAs with enhanced antimetastatic properties and in the analyses of cellular and molecular mechanisms underlying these effects. Regarding novel miRNAs, the strategy is to design appropriate pairs in which one is metastasis-suppressive and the other is metastasis-promotive. Based on the results of numerous experimental studies and reviews, it appears that it is more difficult to obtain miRNAs that contribute to metastasis suppression than those that contribute to metastasis promotion. From this perspective, *Nanog*-activated cancer cells and iPS cells could be innovatively employed to prepare metastasis-suppressive EVs. EVs from *Nanog*-activated cancer X cells are expected to represent a resource that can suppress the metastasis of cancer X cells. On the other hand, EVs from iPS cells are expected to provide a resource that can suppress metastasis in any type of cancer. This simple idea may lead to a useful approach across cancer types.

Given the complexity of the components within EVs, it is extremely difficult to fully elucidate their functional roles and use in stemness-high cells. However, based on the research trends observed in this review, it is expected that various miRNA conditions (types, combinations, concentrations, etc.) will lead to high-performance treatments to reduce metastasis risk to almost zero.

## Figures and Tables

**Table 1:** Candidates for melanoma metastasis-regulating miRNAs and their target genes with high functional association [3,23].

Up or Down	Differentially Analyzed EVs Pair	miRNAs	Target Gene
Up-miRNAs	*Nanog*^+^F10-EVs/F10-EVs	miR-3473b	*Trp53*
		miR-18a-5p	*Hif1a*
			*Esr1*
			*Atm*
		miR-19a-3p	*Rnf11*
	iPS-EVs/*Nanog*^+^F10-EVs	miR-466f-3p	*Ins1*
			*Kitl*
			*Fgf16*
			*Grb2*
			*Malt1*
	*Nanog*^+^colon26-EVs/colon26-EVs	miR-466f-3p	*Ins1*
			*Erbb3*
		miR-122-5p	*Pkm*
Down-miRNAs	*Nanog*^+^F10-EVs/F10-EVs	miR-706	*Cdkn1b*
	iPS-EVs/*Nanog*^+^F10-EVs	miR-342-5p	*Fgf1*
			*Cmklr1*
			*Siglec1*
			*Cd28*
		miR-30a-3p	*Il1f6*
	*Nanog*^+^colon26-EVs/colon26-EVs	miR-22-3p	*Csf1r*
			*Pik3cd*
		miR-145a-5p	*Pdgfd*
		miR-423-3p	*Fgfr2*
		miR-19b-3p	*Gulp1*

**Table 2:** Tumor-related miRNAs in breast cancer and their relationship with tumorigenesis, metastasis, and drug resistance [25].

miRNA Type	miRNAs	Tumorigenesis	Metastasis	Drug Resistance
Oncogenic miRNAs	miR-21	Promotive	Associated	Associated
	miR-155	Promotive	Promotive	Implicated
	miR-10b	Essential for tumorigenesis	Promotive	-
	miR-221	Promotive	Promotive	Promotive
	miR-222	Promotive	Promotive	Promotive
Tumor suppressive miRNAs	miR-34a	Downregulation associated	Downregulation associated	Downregulation associated
	miR-200 family	Key regulators	EMT inhibition	Downregulated
	miR-122	-	-	Regulating
	let-7 family	Downregulated	Downregulated	Downregulated

**Table 3:** Potential roles of miRNAs in stemness-high cells.

Stemness-High Cells and EVs	miRNAs	Potential Role of miRNAs	Reference
ES cells	miR-302a/302a */302b/302b */302c/302c */302d/367	Expressing both in ES cells and embryonic carcinoma	[26]
	miR-154 */200c/368/371/372/373/373 *	Expressing only in ES cells	
	miR-290/291a/291b/292/293/294/295	Used to discriminate pluripotency from early differentiation events	[27]
	miR-145	Upregulating the expression of smooth muscle markers during early differentiation	[28]
	miR-6086/6087/6088/6089/6090	Promotion of endothelial differentiation by downregulation of these miRNAs	[29]
iPS cells	miR-27b	Antagonization of BMP signaling in early differentiation	[30]
	miR-199a	Support of pluripotency by low expression of miR-199a in a feeder layer	[31]
	miR-302	Control of differentiation via repression of DAZAP2	[32]
	miR-302/367	Maintaining pluripotency	[33]
	miR-21	Induction of differentiation into vascular endothelial cells in the presence of VEGF	[34]
	miR-122/375	Promotion of differentiation into hepatocyte-like cells	[35]
	miR-302a-d/369-3p/5/200c	Reprogramming colon cancer	[36]
	miR-133/155/221/34a	Enhancement of cell proliferation of cortical spheroids and promotion of axonal growth	[37]
	miR-302a/302b/148a/21/20a/302d/302c/182/92a-1/92a-2	Top 10 highly expressed miRNAs as evaluation indicators for mass-produced EVs	[38]
MS cells	miR-299-5p	Involved in the suppression of osteogenesis from MS cells when co-cultured with human periodontal ligament cells (HPL cells)	[39]
	miR-101-3p	Involved in the suppression of osteogenesis from MS cells when co-cultured with human gingival fibroblasts (HGF)	[40]
	miR-143/145	Upregulated by TGF-β1 in differentiation into vascular smooth muscle cells (VSMCs)	[41]
	miR-503	Promotion of differentiation into VSMCs via the TGF-β1 pathway	
	miR-222-5p	Suppression of differentiation into VSMCs by downregulation of ROCK2 and α-SMA	
	emiR-200b-3p	Promotion of differentiation into insulin- producing cells from umbilical cord MS cells by targeting ZEB2	[42]
	mR-145	Promotion of differentiation of bone marrow MS cells into smooth muscle cells	[43]
	miR-29b	Promotion of osteogenic differentiation of MS cells via the PTEN/AKT/β-catenin signaling pathway	[44]
	miR-125b	Regulation of osteogenic differentiation of MS cells by targeting Cbfβ	[45]
	miR-34a	Activation of alveolar differentiation by inhibiting TGFβ signaling and activating BPM pathway	[46]
	miRNA-146a/487b-3p	Prognostic markers for chondrogenic differentiation potential of equine MS cells	[47]
	miR-15b-5p (CL1), miR-27b-3p (CL2), miR-18a-5p/7 (CL3), let-7c-5p (CL4), miR-503-5p (CL5)	Relevant to trans-differentiation of MS cells into neurons, categorized into 5 clusters (CL) with different expression time-courses.	[48]

* indicates variant.

**Table 4:** miRNAs activating and regulating macrophages [54].

Macrophage	miRNAs
M1-like activation	miR-125b, miR-127, miR-155
Negative feedback in M1	miR-21, miR-146
M2-like activation	Let-7e, miR-124, miR-223
Negative feedback in M2	miR-23, miR-27, miR-511

**Table 5:** miRNAs associated with innate immune system.

Macrophage [54]	NK Cells [56,57,58,59]	DCs [61,62]
miR-125b	miR-29a/29b/29c	miR-5119
miR-127	miR-17-5p	miR-221
miR-155	miR-338-3p	miR-222
miR-21		miR-155
miR-146		miR-142-3p
let-7e		miR-146a
miR-124		
miR-223		
miR-23		
miR-27		
miR-511		

**Table 6:** Properties and functional roles of metastasis-regulating miRNAs described in references cited as cancer-associated miRNAs and in Section 6 and Section 7.

miRNAs	(P/S) Properties and Functional Roles	Reference
miR-18a-5p	(In cancer cells, P) Exosomal miR-18a-5p secreted from nasopharyngeal carcinoma NPC cells promotes proliferation, migration, invasion, and EMT of NPC cells. Exosomal miR-18a-5p derived from NPC cells targets the anti-proliferative factor BTG3 and promotes angiogenesis by activating the Wnt/β-catenin signaling pathway.	[65]
	(P) Deregulation of the miR-18a-5p/P4HB network contributes to the developmental origin of prostate cancer in offspring of maternal malnutrition.	[66]
miR-19a-3p	(P and S) Identification of eight genes and regulatory mechanisms that influence the prognosis of patients with neuroendocrine tumors (NENs), including miR-19a-3p.	[67]
	(GBP1 is P,S) GBP1 expression induced by INFγ is associated with S in colorectal cancer and prostate cancer, and P in poor prognosis in oral squamous cell carcinoma and ovarian cancer.	[68]
miR-3473b	(P) miR-3473b from lung cancer cell-derived exosomes is taken up by lung fibroblasts and inhibits the function of NFKB inhibitor delta (NFKBID), activating NF-κB signaling and increasing the intrapulmonary colonization of lung tumor cells.	[69]
miR-466f-3p	(S in injury) Among the miRNA cargos enriched in mMSCs-Exo, miR-466f-3p effectively alleviates radiation-induced lung injury via inhibition of the AKT/GSK3β pathway.	[70]
	(P) Downregulation of 466f-3p is effective in maintaining the mesenchymal phenotype of CSCs isolated from medulloblastomas harboring mutations in the Sonic Hedgehog pathway.	[71]
miR-122-5p	(P in disease) Expression of miR-122-5p was significantly upregulated in systemic lupus erythematosus (SLE) exosomes.	[72]
miR-342-5p	(Marker)Circulating exosomal miR-342-5p and miR-574-5p may serve as novel diagnostic biomarkers for early-stage lung adenocarcinoma (LA) patients.	[73]
miR-30a-3p	(S) miR-30a/e-3p expression downregulates both TGF-βR1 and BMPR2 and attenuates the survival and motility of HNSCC.	[74]
miR-145a-5p	(S) Extracellular miR-146a-5p plays important roles in innate immune regulation.	[75]
miR-423-3p	(S) miR-423-3p was highly expressed in hypoxic glioma-derived exosomes (H-GDEs) and played an important role in autophagy, resulting in the activation of normal human astrocytes.	[76]
miR-19b-3p	(S in disease) Upregulation of miR-19b-3p has protective effects against CVB3-induced myocardial injury in mice.	[77]
